# Comparative metabolomic analysis of exudates of microcystin-producing and microcystin-free *Microcystis aeruginosa* strains

**DOI:** 10.3389/fmicb.2022.1075621

**Published:** 2023-01-19

**Authors:** Yuan Zhou, Jun Xu, Hugh J. MacIsaac, Robert Michael McKay, Runbing Xu, Ying Pei, Yuanyan Zi, Jiaojiao Li, Yu Qian, Xuexiu Chang

**Affiliations:** ^1^School of Ecology and Environmental Science, Yunnan University, Kunming, China; ^2^Department of Ecology and Environment of Yunnan Province, Kunming Ecology and Environment Monitoring Station, Kunming, China; ^3^Great Lakes Institute for Environmental Research, University of Windsor, Windsor, ON, Canada; ^4^College of Agronomy and Life Sciences, Kunming University, Kunming, China

**Keywords:** *Microcystis aeruginosa*, untargeted metabolomics, growth phase, differential metabolites, cyanobacterial harmful algal blooms

## Abstract

Cyanobacterial harmful algal blooms (cHABs) dominated by *Microcystis aeruginosa* threaten the ecological integrity and beneficial uses of lakes globally. In addition to producing hepatotoxic microcystins (MC), *M. aeruginosa* exudates (MaE) contain various compounds with demonstrated toxicity to aquatic biota. Previously, we found that the ecotoxicity of MaE differed between MC-producing and MC-free strains at exponential (E-phase) and stationary (S-phase) growth phases. However, the components in these exudates and their specific harmful effects were unclear. In this study, we performed untargeted metabolomics based on liquid chromatography-mass spectrometry to reveal the constituents in MaE of a MC-producing and a MC-free strain at both E-phase and S-phase. A total of 409 metabolites were identified and quantified based on their relative abundance. These compounds included lipids, organoheterocyclic compounds, organic acid, benzenoids and organic oxygen compounds. Multivariate analysis revealed that strains and growth phases significantly influenced the metabolite profile. The MC-producing strain had greater total metabolites abundance than the MC-free strain at S-phase, whereas the MC-free strain released higher concentrations of benzenoids, lipids, organic oxygen, organic nitrogen and organoheterocyclic compounds than the MC-producing strain at E-phase. Total metabolites had higher abundance in S-phase than in E- phase in both strains. Analysis of differential metabolites (DMs) and pathways suggest that lipids metabolism and biosynthesis of secondary metabolites were more tightly coupled to growth phases than to strains. Abundance of some toxic lipids and benzenoids DMs were significantly higher in the MC-free strain than the MC-producing one. This study builds on the understanding of MaE chemicals and their biotoxicity, and adds to evidence that non-MC-producing strains of cyanobacteria may also pose a threat to ecosystem health.

## Introduction

Cyanobacterial harmful algal blooms (cHABs)- often dominated by *Microcystis* spp. are increasing in frequency and severity globally, with further increases predicted coincident with climate change (Harke et al., 2016; Paerl et al., 2016; Huisman et al., 2018; Ho et al., 2019). Cyanobacteria are renowned for their negative impacts on aquatic ecosystems, largely owing to the noxious and harmful, secondary metabolites that they produce and release upon cell lysis (Carmichael and Boyer, 2016; Janssen, 2018; Jones et al., 2021). The ecotoxic effects of cHABs directly and indirectly impact other bacterioplankton, phytoplankton, and zooplankton (Zhang et al., 2009; Chen et al., 2016; Ger et al., 2016; Dias et al., 2017; Wang et al., 2017; Wituszynski et al., 2017; Xu et al., 2019). Toxic effects are also reported in animals and humans (Carmichael and Boyer, 2016; Papadimitriou et al., 2018; Zi et al., 2018; Breinlinger et al., 2021; Cai et al., 2022).

While more than 5,000 studies have been published on production and toxicity of microcystins (MCs; Janssen, 2018), cyanobacteria also produce a wide range of other organic compounds that vary in concentration and toxicity (e.g., aeruginosin, anabaenopeptin, cyanopeptolin, microginin, microviridin, aerucyclamide and retinoic acids; Janssen, 2018; Huang and Zimba, 2019; Yeung et al., 2020). Research on these other toxic compounds of cyanobacteria has been far more limited than that on MCs (Ma et al., 2015; Racine et al., 2019; Jones et al., 2021).

*Microcystis* strains may be characterized as ‘Microcystin-producing’ (MC-producing strain) or ‘Microcystin-free’ (MC-free strain; Davis et al., 2009). It is known that MC-producing strains often coexist with MC-free strains in nature, and their proportions change seasonally (Kurmayer and Kutzenberger, 2003; Lorena et al., 2004; Hu et al., 2016; Islam and Beardall, 2017; Fernanda et al., 2019). Moreover, previous laboratory research suggests that both MC-producing and MC-free strains can be harmful-eliciting damage to mitochondrial function by altering the membrane potential-and that the latter strain may be more toxic than the former (Xu, 2021). Histopathological observations indicate that both MC-free and MC-producing *Microcystis aeruginosa* induce liver cellular impairments in medaka fish, possibly in association with toxic metabolites (Manach et al., 2018). However, information on other toxic metabolites of MC-free strains is lacking.

*Microcystis aeruginosa* is one of the most common *Microcystis* species (Harke et al., 2016). Exudates produced and released by *M. aeruginosa* (MaE) have a greater impact on other organisms than extracts (derived from freeze–thaw treatment or lyophilization) prepared from cultures. For example, MaE have higher, more estrogenic potential than extracts from cells (Sychrova et al., 2012). The aquatic plant *Potamogeton malaianus* was significantly more sensitive to MaE than to extracts (Zheng et al., 2013). Likewise, compared to extracts, MaE had a stronger effect on the structure of the biofilm microbial community on leaves of *Vallisneria natans* (Jiang et al., 2019). MaE also has adverse effects on aquatic animals, such as estrogenic effects in *Daphnia magna* (Xu et al., 2019), and embryonic heart failure and neurotoxicity to early-life stages in fish (Zi et al., 2018; Cai et al., 2022).The synthesis and release of MaE can be influenced by many factors, including different growth stages. Typical growth phases of cyanobacteria include lag, exponential, stationary and decline phases, and toxicity may vary by phase. For example, MaE from exponential growth phase (E-phase) cultures disrupted photosynthesis and induced oxidative stress in submerged macrophytes (Xu et al., 2015), and inhibited growth of green algae and diatoms much more than MaE from stationary growth phase (S-phase) cultures (Wang et al., 2017). Notably, MaE obtained from S-phase cultures had a stronger effect on the mitochondrial membrane potential of *D. magna* than that of E-phase (Xu, 2021).

We hypothesize that MaE contains metabolites whose concentration and toxicity are influenced by strain-type and culture growth phases. We used untargeted metabolomics based on liquid chromatography-mass spectrometry (LC–MS) to identify metabolites coupled with multivariate data analyses to compare metabolome profiles of MaE of MC-producing and MC-free strains in cultures at both E-phase and S-phase (Rinschen et al., 2019; Chen et al., 2020; Zhang et al., 2021). The study was designed to identify, classify and compare the differential metabolites and potentially harmful compounds in the E and S-phase MaE of MC-producing and MC-free strains. We also performed Kyoto Encyclopedia of Genes and Genomes (KEGG) classification on these exudates to identify potential biosynthetic pathways.

## Materials and methods

### Strains cultivation

*Microcystis aeruginosa*, MC-producing (FACHB-905) and MC-free (FACHB-526) strains were provided by the Freshwater Algae Culture Collection of the Institute of Hydrobiology (FACHB-Collection) at the Chinese Academy of Sciences. The two strains originated from Dianchi Lake in Kunming and Dong-hu Lake in Wuhan, respectively. Both Dianchi and Donghu are eutrophic lakes heavily impacted by cHABs (Liu et al., 2016; Yan et al., 2017; Li et al., 2019). Strains were grown in a modified HGZ-145 medium at 25 ± 1°C at 50 μmol quanta m^−2^ s^−1^, with a 12:12 h light–dark cycle, and gently mixed twice daily by hand (Xu, 2021). Strains were cultured in 1,000 ml of nutrient solution in 2,000 ml Erlenmeyer flasks with six biological replicates. Initial inoculation density was 2.0 × 10^6^ cells/ml. One milliliter of each culture was collected under aseptic conditions daily in order to develop a growth curve and identify cell growth phase based on cell density. Culture approaches adopted principles of sterile technique and routine microscopic examination to verify the low abundance of heterotrophic microbiome (Fernanda et al., 2019; Pound et al., 2021).

### Experimental design and sample collection

*Microcystis aeruginosa* cells were counted daily using a hemocytometer and an optical microscope (Olympus, BX51, Japan). E-phase and S-phase cultures of both strains were harvested on days 3 and 35 for MaE analysis.

To obtain MaE, cultures were clarified by centrifugation at 6,000 × *g* for 10 min following which supernatant was filtered through a 0.22 μm glass fiber filter (MiLiMo separation technology limited company, Shanghai, China). After filtration, MaE was flash-frozen using liquid nitrogen, and all samples stored at −80°C for subsequent metabolomics analysis (Pinu et al., 2018). We found the cells remained intact under the microscope at two growth stages, and no turbidity was observed in extracellular exudate during centrifugation.

Hereafter, we refer to MaE of the MC-producing strain collected at E- and S-phases as MCE and MCS, respectively, while that of the MC-free strain are MCFE and MCFS, respectively.

### LC–MS analysis

To a lyophilized 1 ml exudates sample, we added 500 μl acetonitrile: methanol: H_2_O (2:2:1, containing isotopically-labelled internal standard mixture), following which we vortexed for 30 s, sonicated for 10 min in an ice-water bath, and incubated for 1 h at −40°C to precipitate proteins. The sample was then clarified by centrifugation at 10,000 × *g* for 15 min at 4°C. The quality control (QC) sample was prepared by mixing an equal aliquot of the supernatants from all the samples. During the pre-treatment process, samples were added with three isotopically-labelled internal standards (HPLC purity, Sigma Aldrich) in each of the positive and negative ion modes for repeatability and availability.

Supernatant was analyzed by Ultra High Pressure Liquid Chromatography (UHPLC)-Orbitrap MS (Thermo Fisher Scientific, MA, USA). UHPLC separation was performed using ACQUITY UPLC BEH amide column (2.1 mm × 100 mm, 1.7 μm). The mobile phase consisted of 25 mmol/l ammonium acetate and ammonia hydroxide in water (phase A) and acetonitrile (phase B). The auto-sampler temperature was 4°C, and the injection volume was 3 μl. Q Exactive mass spectrometer (Thermo, Massachusetts, USA) was used to acquire MS/MS spectra on information-dependent acquisition (IDA) mode in the control of the acquisition software (Xcalibur, Thermo), and acquisition from m/z 100 to 1,100. At different collision energy (10/30/60 NCE), the MS/MS spectra of QC samples were obtained off the top 10 precursor ions.

### Data processing

The acquired raw data were converted to the mzXML format using ProteoWizard and processed. After raw data pre-processing, peak detection, extraction, alignment and integration, metabolites were annotated by an in-house database (Biotree database, Biotechnology Co., Ltd., Shanghai, China). The database was built with available commercial standard compounds and existing public mass spectrometry databases, including HMDB^1^, MoNA^2^ and METLIN.^3^ Metabolites were identified by strict criteria steps (Liang et al., 2020; Shen et al., 2020), including comparison of accurate mass (m/z, ±10 ppm), MS/MS spectra similarity score (considers both fragments and intensities), and isotope distribution. The identification of compounds met the level 1and 2 according to the Metabolomics Standards Initiative (Alexandra et al., 2016; Viant et al., 2017).

Quantitative analysis and relative concentration were calculated by the internal standard normalization for peak area method (Sun et al., 2019). Metabolites were quantified by the internal standard with the lowest RSD value. Positive and negative ion mode metabolites were quantitative separately. Retention time and abundance of internal standard in QC and blank samples were stable. The data acquisition stability and accuracy of the method meet the requirements of metabolomic studies (Supplementary Figure S3; Broadhurst et al., 2018). Metabolite peaks present in <50% of group samples were removed from the subsequent analysis, and missing values were imputed with the minimal peak value of the metabolomics dataset (Sun et al., 2019; Liu et al., 2021).

### Statistical analysis

Student’s *t*-test was used to compare growth rate of the two strains. Data were analyzed usingGraphPad Prism 8.0 (GraphPad Software, Inc., San Diego, CA). Principal component analysis (PCA) and orthogonal partial least squares discriminant analysis (OPLS-DA) were used to visualize the differences between and within groups. PCA and OPLS-DA were performed using SIMCA14.1 (Sartorius AG, Gottingen, Germany). Differential metabolites (DMs) were determined by variable importance in projection (VIP) from the OPLS-DA model and fold change (FC; VIP score ≥ 1, absolute Log2FC ≥ 1; Wang et al., 2020; Zhang et al., 2021). Hierarchical cluster analysis, Venn and volcano maps were produced using R version 3.6.3 (pheatmap package, VennDiagram, ggpubr, ggthemes packages). The Kyoto Encyclopedia of Genes and Genomes (KEGG) database (organism-dependent:^3^ *M. aeruginosa*) was used to search metabolic pathways. DMs pathways were analyzed according to the type of KEGG pathway.

## Results

### Growth of MC-producing and MC-free strains

**Figure 1:**
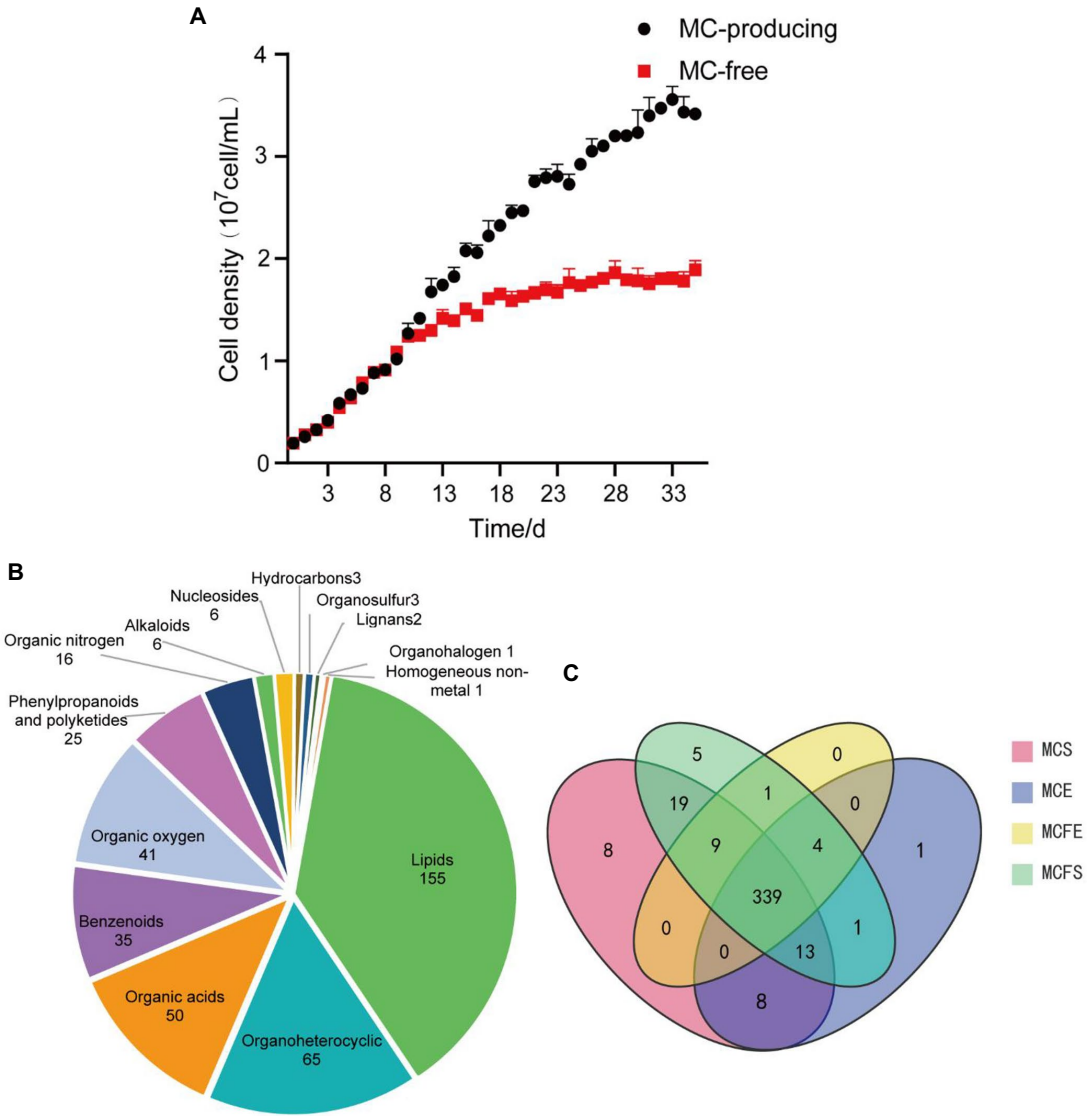
Growth curves of the MC strain and MC-free strain, data are presented as means±standard deviation (*n* = 6; **A**). Pie diagram showing classification of 409 total metabolites identified in MaE **(B)**; Venn diagram of metabolites distribution in four groups, with numbers representing metabolites in common **(C)**. MCE, MC-producing strain at exponential phase; MCFE, MC-free strain at exponential phase; MCS, MC-producing strain at stationary phase; MCFS, MC-free strain at stationary phase.

Growth curves demonstrate that MC-producing and MC-free strains had the same growth rate and similar cell density from day 1 to 10 (Figure 1A). The growth rate of the MC-free strain started to decrease after day 11. By day 35 and cultures in stationary phase, the MC-producing strain achieved a greater cell density than the MC-free strain cultures (*P*<0.05). Cell density of the MC-producing and MC-free strains at E-phase (day 3) were 4.12 × 10^6^ cells/mL and 4.11 × 10^6^ cells/mL, respectively, and 3.42 × 10^7^ cells/mL and 1.87 × 10^7^ cells/mL at S-phase (day 35).

### Metabolite classification and profiling

In total, 409 metabolites were identified among the four MaE groups. These metabolites were grouped into 14 categories at the superclass level with Chemical Taxonomy of HMDB (Figure 1A), mainly including lipids, organoheterocyclic compounds, organic acid, benzenoids, organic oxygen, phenylpropanoids, organic nitrogen, alkaloids, nucleosides, hydrocarbons, organosulfur, lignans, organohalogen compounds and homogeneous non-metals. Detailed information on these metabolites is highlighted in Supplementary Table S1.

The majority (339) of these compounds overlapped in the four groups, indicating that 83% of the metabolites were common (Figure 1B). The relative concentrations of metabolites in MaE in the four groups are presented in Table 1. In terms of total metabolite relative concentrations, the MC-free strain was 10.9% higher than that of the MC-producing strain at E-phase. The accumulation of some metabolites such as benzenoids, hydrocarbons, lipids, nucleosides, organic oxygen and organoheterocyclic compounds were higher in the MC-free strain than in the MC-producing strain at E-phase. However, at S-phase, total metabolites of the MC-producing strain were 24.7% higher than that of the MC-free strain, mainly owing to the higher content of lipids, organic acids, organic oxygen and organoheterocyclic compounds. Total metabolites relative concentration of MaE was higher in S-phase than in E-phase in both strains.

**Table 1:** Relative concentration of metabolites in MaE.

Biochemical categories	Relative concentration index			
	MCE	MCFE	MCS	MCFS
Alkaloids	8.38	6.77	8.59	9.36
Benzenoids	34.2	35.82	44.98	48.58
Hydrocarbons	0.27	0.3	0.29	0.27
Lignans	0.59 × 10^−4^	0.45 × 10^−4^	0.072	0.023
Lipids	25.2	29.07	67.47	45.56
Nucleosides	0.02	0.05	2.2	0.79
Organic acids	6.99	6.81	19.64	11.89
Organic nitrogen	1.69	1.8	2.54	1.77
Organic oxygen	38.55	48	47.18	40.73
Organoheterocyclic	7.88	8.19	22.45	14.31
Organosulfur	0.019	0.017	0.21	0.024
Phenylpropanoids	1.13	1.09	2.96	2.01
Total	124.3	137.9	218.6	175.3

MCE, MC-producing strain at exponential phase; MCFE, MC-free strain at exponential phase; MCS, MC-producing strain at stationary phase; MCFS, MC-free strain at stationary phase.

**Figure 2:**
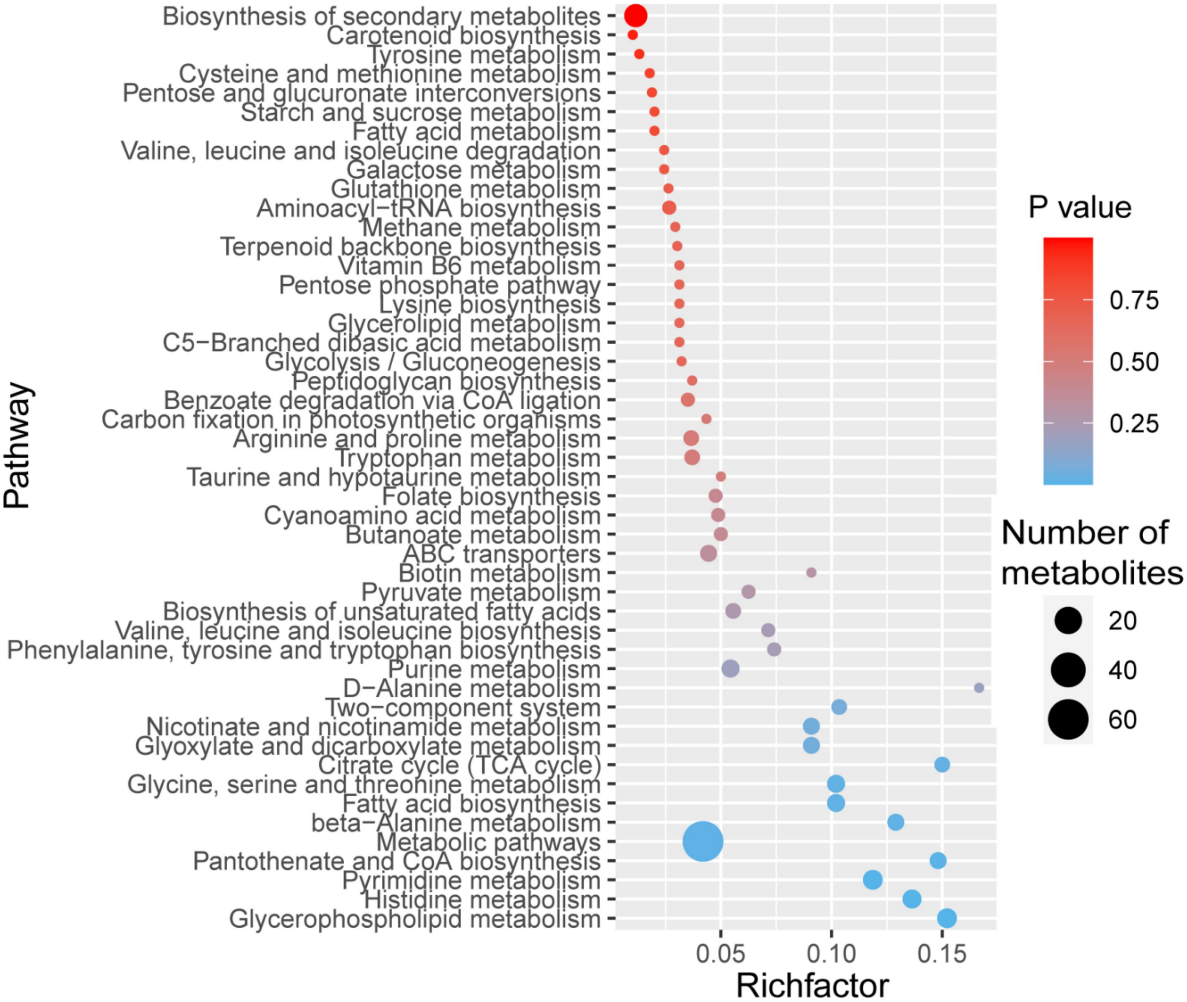
Bubble diagram showing KEGG pathway annotation covered by 409 metabolites. The x-axis indicates the scale of the “Rich factor” (the ratio of the number of metabolites in the corresponding pathway to the total metabolites annotated by the pathway detection) of the pathway in topological analysis, while the y-axis presents individual pathways. The color of the bubble indicates the *p* value of enrichment analysis with darker colors having a lower p value and greater enrichment. Size of the bubble is proportional to the number of metabolites in this pathway.

We identified 50 pathways in the KEGG database for the 409 MaE metabolites in the four groups (Figure 2). These metabolites were involved in lipid pathways (such as metabolism of glycerophospholipid, glycerolipid, fatty acid, biosynthesis of unsaturated fatty acids), carbohydrate metabolism (such as pentose phosphate pathway, starch and sucrose metabolism, citrate cycle), amino acid synthesis (such as histidine metabolism, glycine, serine and threonine metabolism, phenylalanine, tyrosine and tryptophan biosynthesis, valine, leucine and isoleucine biosynthesis, arginine and proline metabolism), and secondary metabolites pathways (such as benzoate degradation *via* CoA ligation, folate biosynthesis, carotenoid biosynthesis, terpenoid backbone biosynthesis, nicotinate and nicotinamide metabolism, cyanoamino acid metabolism).

### Multivariate statistical analysis of metabolites

**Figure 3:**
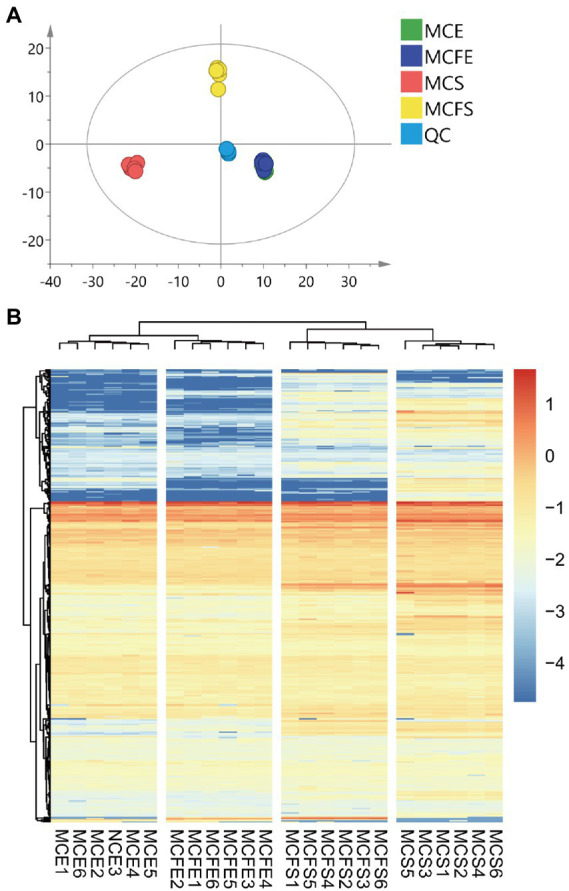
Principal component analysis (PCA) of metabolic profiles in all samples, with six replicates per treatment **(A)**; Cluster heatmap of metabolite content in different samples **(B)**. The x-axis represents six replicate cultures of each of the four treatment groups, the y-axis represents individual metabolites of the groups. Color blocks represent the relative concentration of metabolites at the corresponding positions. MCE, MC-producing strain at exponential phase; MCFE, MC-free strain at exponential phase; MCS, MC-producing strain at stationary phase; MCFS, MC-free strain at stationary phase.

Principal component analysis (PCA) of metabolites from different strains and growth phases revealed that the first and second principal components PC [1] and PC [2] explained 34.3 and 15.2% of total variation, respectively (Figure 3A). PCA results demonstrated that metabolites of the two strains overlapped almost completely during E-phase, but were clearly separated during S-phase, indicating metabolic shifts during growth in both strains. Variation between replicates was greater for S-phase cultures than that observed with E-phase cultures.

Hierarchical cluster analysis (HCA) revealed differences in metabolite profiling between strains and growth phases (Figure 3B). Regardless of strain, metabolites from E-phase and S-phase clustered together, indicating greater homogeneity among growth phases than among strains. Further, most metabolites exhibited higher relative concentrations in stationary than exponential growth phase.

The orthogonal partial least squares discriminant analysis (OPLS-DA) was performed to reveal the differential metabolites (DMs) for the different strains at the same phase and the same strain at different phases (Supplementary Figure S1). Values of *R*^2^*Y* and *Q*^2^ from the permutation test for the OPLS-DA model were higher than their original values, indicating good quality of each supervised model without overfitting (Supplementary Figure S2).

### Differential metabolites identification and analysis

#### DMs of different strains in the same growth phase

**Figure 4:**
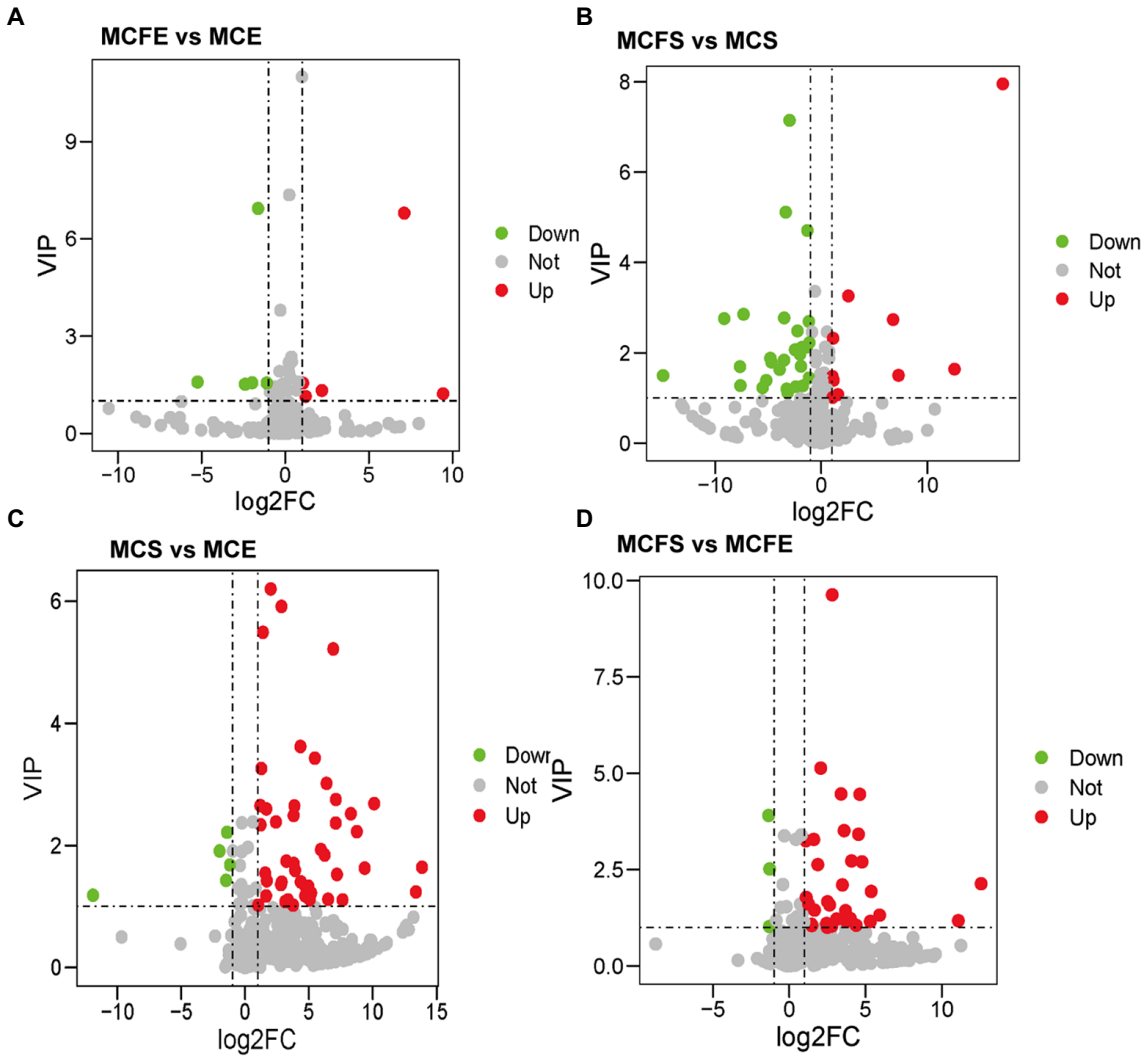
Differential metabolites in pairwise comparison among four MaE groups: volcano plots of differential metabolites in MCFE vs. MCE **(A)**; MCFS vs. MCS **(B)**; MCS vs. MCE **(C)**; MCFS vs. MCFE **(D)**. MCE, MC-producing strain at exponential phase; MCFE, MC-free strain at exponential phase; MCS, MC-producing strain at stationary phase; MCFS, MC-free strain at stationary phase.

There were 10 DMs in exponential cultures MCFE vs. MCE (5 each up-regulated and down-regulated, Figure 4A), and 38 DMs in stationary cultures MCFS vs. MCS (10 up-regulated and 28 down-regulated, Figure 4B). Thus, the total number of DMs at exponential phase was much less than that at stationary phase. Four DMs of different strains at same growth phase overlapped, including 7-ketocholesterol, choline, sinapyl alcohol and [8]-Dehydrogingerdione. 7-ketocholesterol and sinapyl alcohol in the MC-free strain were significantly higher than those of the MC-producing strain at both growth phases.

**Figure 5:**
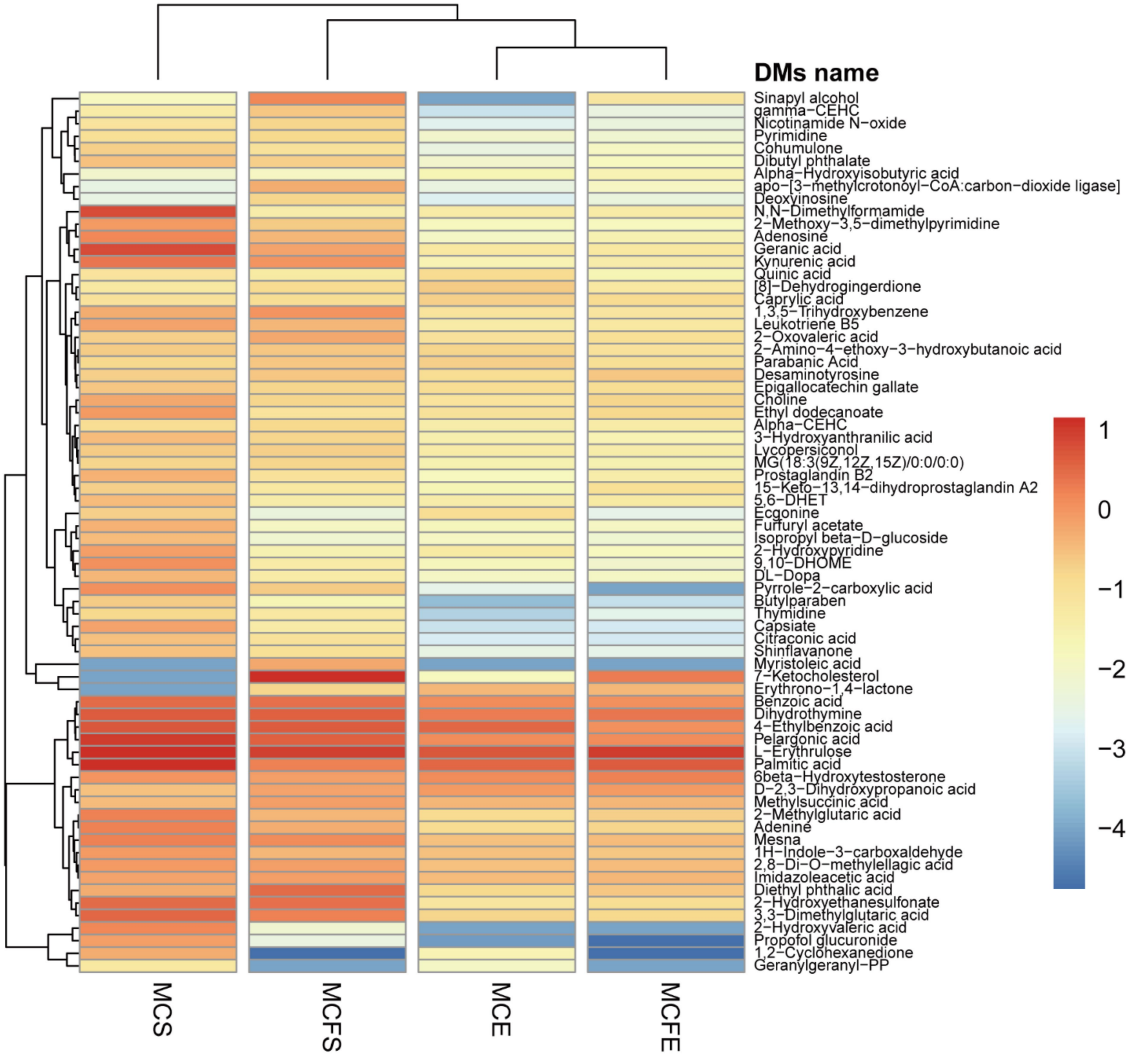
Heatmap for differential metabolites based upon hierarchical clustering of the four groups. The *x*-axis represents the four experimental groups, the *y*-axis the differential metabolites. The color blocks represent the relative concentration of metabolites at the corresponding positions. MCE, MC-producing strain at exponential phase; MCFE, MC-free strain at exponential phase; MCS, MC-producing strain at stationary phase; MCFS, MC-free strain at stationary phase.

DMs with similar variation trends in concentration were positioned closer together on the HCA heat map (Figure 5). In terms of clustering between groups, the similarity of the two strains in E-phase was greater than that in S-phase. Relative concentration of most DMs in stationary phase was higher than that in E-phase for both strains.

#### DMs of same strain in different growth phases

There were 50 DMs in MCS vs. MCE (45 up-regulated and 5 down-regulated, Figure 4C), and 36 DMs in MCFS vs. MCFE (33 up-regulated and 3 down-regulated, Figure 4D). The total number of DMs of the MC-producing strain were greater than those of the MC-free strain. Growth phase significantly affected metabolites, as most lipids, organoheterocyclic compounds, benzenoids, organic acids, phenylpropanoids and nucleosides were significantly up-regulated in the S-phase. Secondary metabolites, such as flavonoids, phenylpropanoids, benzene and substituted derivatives, indoles and lactones were significantly up-regulated in S-phase cultures. Nine DMs overlapped and displayed the same change trend, that was up-regulated during the S-phase for both strains, including 1,3,5-trihydroxybenzene, 2-hydroxyethanesulfonate, 3-hydroxyanthranilic acid, adenine, adenosine, kynurenic acid, mesna, pyrrole-2-carboxylic acid and sinapyl alcohol. We putatively identify these overlapped metabolites as key growth phase-related metabolites of *M. aeruginosa*. Detailed information on DMs is provided in Supplementary Table S2.

### Metabolic pathway analysis of DMs

#### DMs pathway of different strains in the same growth phase

**Figure 6:**
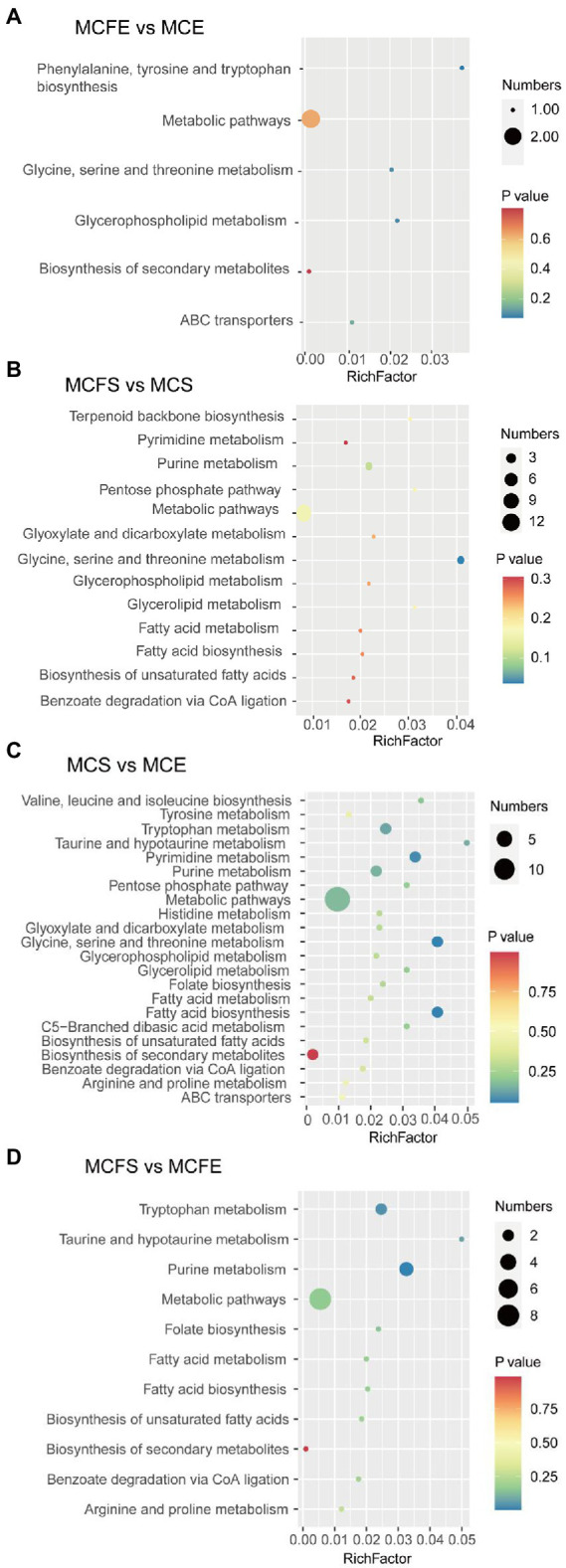
Bubble diagram of KEGG pathway annotation covered by differential metabolites. The x-axis indicates the scale of the “Rich factor” (the ratio of the number of differential metabolites in the corresponding pathway to the total metabolites annotated by the pathway detection), while the y-axis presents individual pathways identified. The color of the bubble indicates the p value of enrichment analysis with darker colors having a lower value and more significant enrichment. Size of the bubble is proportional to the number of metabolites in this pathway. Bubble diagrams of MCFE vs. MCE **(A)**; MCFS vs. MCS **(B)**; MCS vs. MCE **(C)**; MCFS vs. MCFE **(D)**. MCE, MC-producing strain at exponential phase; MCFE, MC-free strain at exponential phase; MCS, MC-producing strain at stationary phase; MCFS, MC-free strain at stationary phase.

Differential metabolites were linked to metabolic pathways in the KEGG database. In the MCFE vs. MCE group, DMs mapped to six pathways (Figure 6A), including biosynthesis of secondary metabolites, glycine, serine and threonine metabolism, glycerophospholipid metabolism, ABC transporters and aromatic amino acid biosynthesis. In the MCFS vs. MCS comparison, DMs were mapped to 13 pathways (Figure 6B), including pyrimidine metabolism, purine metabolism, lipids and amino acid metabolisms, and benzoate degradation *via* CoA ligation.

#### DMs pathway of same strain in the different growth phases

In the MCS vs. MCE group, DMs mapped into 22 pathways (Figure 6C), including biosynthesis of secondary metabolites, amino acid metabolism, pyrimidine and purine metabolism and lipid metabolites. In the MCFS vs. MCFE group, the DMs were mapped to 11 pathways (Figure 6D), including biosynthesis of secondary metabolites, amino acid metabolism, purine metabolism, folate biosynthesis, lipid metabolism and benzoate degradation *via* CoA ligation.

**Figure 7:**
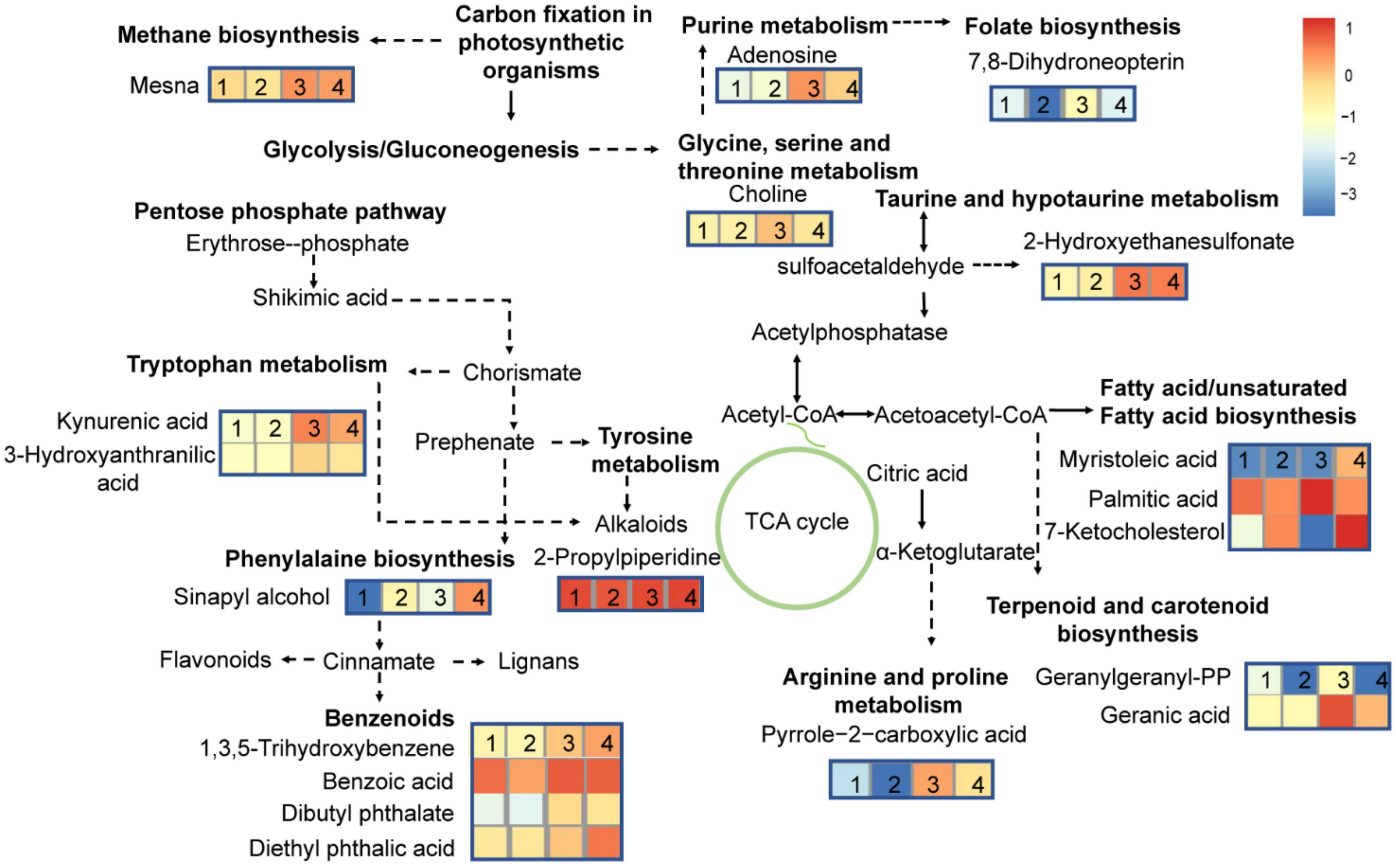
Differential metabolites pathways in MC-producing and MC-free strains harvested at exponential- and stationary-growth phases. The numbers 1, 2, 3, and 4 represent MCE, MCFE, MCS, MCFS, respectively. MCE, MC-producing strain at exponential phase; MCFE, MC-free strain at exponential phase; MCS, MC-producing strain at stationary phase; MCFS, MC-free strain at stationary phase.

At different growth phases, the MC-producing strain had 11 more DMs pathways than the MC-free strain. These pathways were mainly involved in lipids and amino acid metabolism. To further highlight the changes in the metabolic pathway induced during growth stage and strain, a metabolic pathway map was generated based on the DMs (Figure 7). DMs pathways were mainly focused on lipids biosynthesis and their downstream pathways, biosynthesis of secondary metabolites, and amino acids biosynthesis pathways.

## Discussion

Cyanobacteria produce many more potentially harmful metabolites aside from the classic toxins such as microcystins (Huang and Zimba, 2019; Ferreira et al., 2021). In this study, we found that exudate mixtures of *M. aeruginosa* contain a large number of lipids, organoheterocyclics, organic acids, benzenoids, organic oxygen compounds, phenylpropanoids and organic nitrogen metabolites. Amongst lipid and organoheterocyclics compounds detected were a number whose toxicologic effects have been established (Table 2). Some organic oxygen compounds, such as carbonyl compounds and ethers, are also toxic. For example, carbonyl compounds were potential mutagens and carcinogens (Vilma et al., 2006), and ethers had antibacterial activity and neurotoxic characteristics (Suyama et al., 2010). Alkaloids (harmala and tropane alkaloids) have pharmacological and therapeutic effective (Moloudizargari et al., 2013; Kathrin and Oliver, 2019). Zi et al. (2022) screened nine neurotoxic compounds, including lysoPC (16:0), 2-acetyl-1-alkyl-sn-glycero-3phosphocholine, egonol glucoside, polyoxyethylene monoricinoleate, and phytosphingosin from MaE by using machine learning and molecular docking methods. Toxic effects of MaE on organisms are likely to result from the combined effect of these mixtures (Dias et al., 2017; Manach et al., 2018).

**Table 2:** Main compounds identified and their toxicologic effects.

Name	Target	Toxicity type	Mode of action	References
Pelargonic acid	Broadleaf and grass weeds	Growth inhibition	-	Webber and Shrefler (2006)
Goyaglycoside	Cancer cells	Cytotoxicity	-	Wang et al. (2012)
Phloroglucinol	Pathogenic fungi, bacteria	Growth inhibition	-	Haas and Keel (2003); Abdel-Ghany et al. (2016)
Geranic acid	Phytopathogen	Antifungal	-	Mi et al. (2014)
Gingerol	Mammary carcinoma	Growth inhibition	Apoptosis	Bernard et al. (2017)
Phyto-sphingosine	Watermelon Fusarium oxysporum	Growth inhibition	-	Li et al. (2020)
	CNE-2 cells	Cytotoxicity	Mitochondria-mediated apoptosis	Li et al. (2022)
3-Amino-1,4-dimethyl-5H-pyrido[4,3-b] indole	Rat Splenocytes	Cytotoxicity	Apoptosis and Necrosis	Hashimoto et al. (2004)
Nicotine	Nerve, blood vessel	Neurotoxicity, atherosclerotic lesions	Autonomic imbalance, endothelial dysfunction and coronary blood flow dysregulation	Adamopoulos et al. (2008)
3-Hydro-xyanthranilic acid	THP-1 and U937 cells		Apoptosis	Morita et al. (2001)
Palmitic acid	Multiple myeloma cells	Growth inhibition	Apoptosis	Nagata et al. (2015)

We observed an orchestrated elevation of some differential metabolites (DMs) in a MC-free strain compared with a MC-producing strain, including 7-ketocholesterol, sinapyl alcohol, myristoleic acid and diethyl phthalic acid. Xu (2021) revealed that MaE was toxic to mitochondrial membranes in *D. magna*, and the MC-free strain was more toxic to mitochondrial membrane than a MC-producing strain, and toxicity effects were stronger in S-phase than E-phase cultures. Additionally, metabolic pathways associated with benzenoids biosynthesis (e.g., phenylalaine biosynthesis,benzoate degradation *via* CoA ligation) were significantly up-regulated in the MC-free strain (Table 1; Figure 7). We suspect that these chemicals in the MC-free strain are linked to mitochondrial membrane damage. 7-Ketocholesterol can activate apoptosis, autophagy and induced mitochondrial damage (Gabriella et al., 2006; Lee et al., 2007; Ghzaiel et al., 2021), in turn causing cellular damage *via* multiple stress-response pathways (Anderson et al., 2020). Sinapyl alcohol exhibited significant cytotoxic activities against human tumor cell lines (Zou et al., 2006; Lee et al., 2015). Myristoleic acid as one of the cytotoxic components, induces mixed cell death of apoptosis and necrosis in LNCaP cells (Kazuhiro et al., 2001). Diethyl phthalic acid belongs to the group of phthalates which are widely applied as plasticizers and solvents in the chemical industry. Phthalates can be endocrine disrupting chemicals and they exhibit both toxicity and bioaccumulation (Sun et al., 2012). Phthalates are also produced by marine algae, with abundance varying among species (Chen, 2004; Namikoshi et al., 2006).

Despite the same culture conditions and initial cells density, MC-producing cultures accumulated more cells and higher concentrations of most primary and secondary metabolites than the MC-free cultures at S-phase (Table 1). Compared to the E-phase, both strains in S-phase had higher numbers and abundance of differential metabolites (DMs) of lipid, organoheterocyclic compounds and benzenoids compounds, indicating that growth phase significantly affects metabolites more than strain type. The dynamic accumulation of metabolites is largely determined by growth processes, as is variation in secondary metabolites (Zhang et al., 2021; Guo et al., 2022). Results of the analysis of DMs and pathways suggest that lipid metabolism and biosynthesis of some amino acids correlate more closely with growth phase than by strain (Figure 6), suggesting that some secondary metabolites—such as alkaloids, sulfide and benzenoids derived from tyrosine metabolism, taurine and hypotaurine metabolism and phenylalaine metabolism, respectively—accumulated during the S-phase (Tiago et al., 2017; Cao et al., 2020). An understanding of the metabolites accumulated in MaE and the dynamic changes in metabolites during exponential and stationary growth phases is essential for assessing the toxicity of compounds and would also provide a basis for subsequent research on *M. aeruginosa* of MC-producing and MC-free strains. *Microcystis* colony formations and other ecological factors, could differ between laboratory and field conditions (Xiao et al., 2018), thus we propose further attention be given to cyanobacterial compounds in relation to biotic and abiotic factors in the field. At the same time, we propose that water quality monitoring guidance consider the different growth stages that may occur in cyanobacterial blooms as well as potential ecological risks associated with MC-free strains and the toxic compounds that they produce.

## Conclusion

*Microcystis aeruginosa* exudates (MaE) contain a large number of lipids, organoheterocyclics compounds, organic acids, benzenoids, organic oxygen compounds, phenylpropanoids and organic nitrogen metabolites. Clear distinctions existed between metabolites of different growth phases, which clearly exceeded differences amongst strains. Some metabolites such as benzenoids, lipids, organic oxygen and organoheterocyclic compounds were higher in the MC-free strain than the MC-producing strain at E-phase. The MC-producing strain reached higher cell density and accumulated more total metabolites than the MC-free strain at S-phase. Some metabolites with known cytotoxicity, apoptosis-inducing effects, neurotoxicity and reproductive toxicity were detected in both strains. This study expands awareness of the metabolites and toxicity of *M. aeruginosa* at different growth phases and across strains, and adds to growing recognition that cyanobacteria can produce numerous compounds with potentially harmful effects on aquatic life.

## Data availability statement

We have registered an account on Metabolightes website (MTBLS6603). The metabolite data is being uploaded for validation.

## Author contributions

XC and HM designed the study. JX, YZh, YP, and YZi collected samples and conducted the experiments. YZh analyzed and interpreted the data. XC, HM, RM, RX, YQ, and JL revised the manuscript. All authors contributed to the article and approved the submitted version.

## Funding

This research was supported by funds from the National Natural Science Foundation of China (NSFC)—Yunnan Joint Key Grant (No. U1902202), Yunnan Provincial Science and Technology Department grants (2019FA043; 2018BC002; and 202101AU070078), and Great Lakes Fishery Commission (2020_MAC_440940).

## Conflict of interest

The authors declare that the research was conducted in the absence of any commercial or financial relationships that could be construed as a potential conflict of interest.

## Publisher’s note

All claims expressed in this article are solely those of the authors and do not necessarily represent those of their affiliated organizations, or those of the publisher, the editors and the reviewers. Any product that may be evaluated in this article, or claim that may be made by its manufacturer, is not guaranteed or endorsed by the publisher.

## Footnotes

^1^ https://hmdb.ca

^2^ https://mona.fiehnlab.ucdavis.edu/

^3^ https://metlin.scripps.edu/

^3^ www.kegg.jp/kegg
