# Corrigendum: Comparative metabolomic analysis of exudates of high-microcystin-producing and low-microcystin-producing *Microcystis aeruginosa* strains

**DOI:** 10.3389/fmicb.2023.1333121

**Published:** 2024-01-10

**Authors:** Yuan Zhou, Jun Xu, Hugh J. MacIsaac, Robert Michael McKay, Runbing Xu, Ying Pei, Yuanyan Zi, Jiaojiao Li, Yu Qian, Xuexiu Chang

**Affiliations:** ^1^School of Ecology and Environmental Science, Yunnan University, Kunming, China; ^2^Department of Ecology and Environment of Yunnan Province, Kunming Ecology and Environment Monitoring Station, Kunming, China; ^3^Great Lakes Institute for Environmental Research, University of Windsor, Windsor, ON, Canada; ^4^College of Agronomy and Life Sciences, Kunming University, Kunming, China

**Keywords:** *Microcystis aeruginosa*, untargeted metabolomics, growth phase, differential metabolites, cyanobacterial harmful algal blooms

In our study, we classified *Microcystis aeruginosa* strain FACHB-526 as microcystin-free based upon its classification at the time of purchase by the Freshwater Algae Culture Collection of the Institute of Hydrobiology at the Chinese Academy of Sciences (FACHB). Following publication of our study, we sought to verify the status of the strain using LC-MS/MS and determined that, in fact, exudates did contain microcystin-LR (MC-LR). Concentrations of MC-LR in exudates of the FACHB-526 strain for exponential and stationary phase cultures were 2.04 and 7.90 ng•mL^−1^, respectively, whereas our microcystin-producing strain (FACHB-905) they were 3.79 and 17.42 ng•mL^−1^, respectively (Xu et al., 2023). Therefore, our study should be considered a comparative analysis of two strains producing low (FACHB-526) and high (FACHB-905) concentrations of MC-LR. The terms “microcystin-producing” and “microcystin-free” have been replaced throughout the article with “high-microcystin-producing (high-MC-producing)” and “low-microcystin-producing (low-MC-producing),” respectively.

In the published article, there was an error in the article title. Instead of “*Comparative metabolomic analysis of exudates of microcystin-producing and microcystin-free Microcystis aeruginosa strains*,” it should be “*Comparative metabolomic analysis of exudates of high-microcystin-producing and low-microcystin-producing Microcystis aeruginosa strains*.”

In addition, the reference “Xu, J., Chang, X., MacIsaac, H. J., Zhou, Y., Li, J., Wang T., et al. (2023). Is a lower-toxicity strain of *Microcystis aeruginosa* really less toxic? *Aquatic Toxicology*, 263, 106705. 10.1016/j.aquatox.2023.106705” was not included in the published article. This has added to the reference list and a citation for it has now been inserted in **Introduction**, paragraph 1. The corrected sentence reads as follows:

“Moreover, laboratory research suggests that both high MC-producing and low MC-producing strains can be harmful-eliciting damage to mitochondrial function by altering the membrane potential-and that the latter strain may be more toxic than the former (Xu, 2021; Xu et al., 2023).”

The citation has also been inserted in **Discussion**, paragraph 2, which reads as follows:

“Xu et al. (2023) revealed that MaE was toxic to mitochondrial membranes in *D. magna*, and the lower-MC strain was more toxic to mitochondrial membrane than high-MC strain, and toxicity effects were stronger in S-phase than E-phase cultures.”

Furthermore, in the published article there were errors in the captions for Figures 1, 3, 4, 5, 6, 7, and Table 1. Due to the re-naming of the strains, the terms “microcystin-producing” and “microcystin-free” have been replaced throughout the article with “high-microcystin-producing (high-MC-producing)” and “low-microcystin-producing (low-MC-producing),” respectively. The corrected legends appear below.

“**Figure 1**. Growth curves of the high and low-MC producing strains, data are presented as means ± standard deviation [*n* = 6; **(A)**]. Pie diagram showing classification of 409 total metabolites identified in MaE **(B)**; Venn diagram of metabolites distribution in four groups, with numbers representing metabolites in common **(C)**. MCE, high-MC-producing strain at exponential phase; MCFE, low-MC-producing strain at exponential phase; MCS, high-MC-producing strain at stationary phase; MCFS, low-MC-producing strain at stationary phase.

**Figure 3**. Principal component analysis (PCA) of metabolic profiles in all samples, with six replicates per treatment **(A)**; Cluster heatmap of metabolite content in different samples **(B)**. The x-axis represents six replicate cultures of each of the four treatment groups, the y-axis represents individual metabolites of the groups. Color blocks represent the relative concentration of metabolites at the corresponding positions. MCE, high-MC-producing strain at exponential phase; MCFE, low-MC-producing strain at exponential phase; MCS, high-MC-producing strain at stationary phase; MCFS, low-MC-producing strain at stationary phase.

**Figure 4**. Differential metabolites in pairwise comparison among four MaE groups: volcano plots of differential metabolites in MCFE vs. MCE **(A)**; MCFS vs. MCS **(B)**; MCS vs. MCE **(C)**; MCFS vs. MCFE **(D)**. MCE, high-MC-producing strain at exponential phase; MCFE, low-MC-producing strain at exponential phase; MCS, high-MC-producing strain at stationary phase; MCFS, low-MC-producing strain at stationary phase.

**Figure 5**. Heatmap for differential metabolites based upon hierarchical clustering of the four groups. The x-axis represents the four experimental groups, the y-axis the differential metabolites. The color blocks represent the relative concentration of metabolites at the corresponding positions. MCE, high-MC-producing strain at exponential phase; MCFE, low-MC-producing strain at exponential phase; MCS, high-MC-producing strain at stationary phase; MCFS, low-MC-producing strain at stationary phase.

**Figure 6**. Bubble diagram of KEGG pathway annotation covered by differential metabolites. The x-axis indicates the scale of the “Rich factor” (the ratio of the number of differential metabolites in the corresponding pathway to the total metabolites annotated by the pathway detection), while the y-axis presents individual pathways identified. The color of the bubble indicates the *P*-value of enrichment analysis with darker colors having a lower value and more significant enrichment. Size of the bubble is proportional to the number of metabolites in this pathway. Bubble diagrams of MCFE vs. MCE **(A)**; MCFS vs. MCS **(B)**; MCS vs. MCE **(C)**; MCFS vs. MCFE **(D)**. MCE, high-MC-producing strain at exponential phase; MCFE, low-MC-producing strain at exponential phase; MCS, high-MC-producing strain at stationary phase; MCFS, low-MC-producing strain at stationary phase.

**Figure 7**. Differential metabolites pathways in high-MC-producing and low-MC-producing strains harvested at exponential-and stationary-growth phases. The numbers 1, 2, 3, and 4 represent MCE, MCFE, MCS, MCFS, respectively. MCE, high-MC-producing strain at exponential phase; MCFE, low-MC-producing strain at exponential phase; MCS, high-MC-producing strain at stationary phase; MCFS, low-MC-producing strain at stationary phase.

**Table 1**. Relative concentration of metabolites in MaE. MCE, high-MC-producing strain at exponential phase; MCFE, low-MC-producing strain at exponential phase; MCS, high-MC-producing strain at stationary phase; MCFS, low-MC-producing strain at stationary phase.”

In the published article, there was also an error in Figure 1 as published. Due to the re-naming of the strains, Figure 1A has been modified. The corrected Figure 1 and its caption appear below.

**Figure 1:**
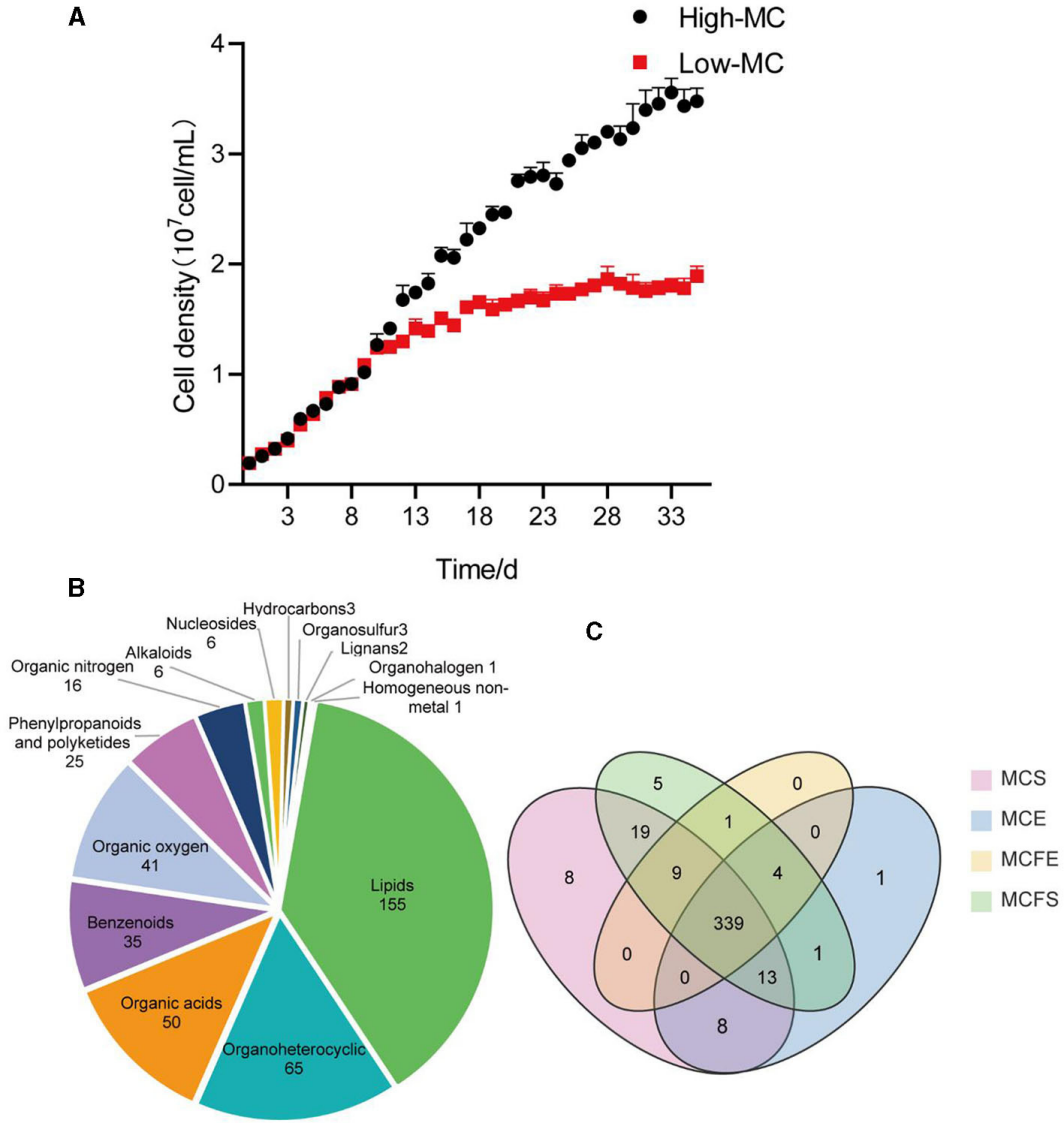
Growth curves of the high and low-MC-producing strains, data are presented as means ± standard deviation [*n* = 6; **(A)**]. Pie diagram showing classification of 409 total metabolites identified in MaE **(B)**; Venn diagram of metabolites distribution in four groups, with numbers representing metabolites in common **(C)**. MCE, high-MC-producing strain at exponential phase; MCFE, low-MC-producing strain at exponential phase; MCS, high-MC-producing strain at stationary phase; MCFS, low-MC- producing strain at stationary phase.

In the published article, there were numerous errors, as the terms “microcystin-producing” and “microcystin-free” have been replaced throughout the article with “high-microcystin-producing (high-MC-producing)” and “low-microcystin-producing (low-MC-producing),” respectively. These terms have been replaced in the following sections of the article:

“**Abstract**, 19–41.
**Introduction**, Paragraph 3, 62–72, paragraph 5, 94–98.
**Materials and methods**, “*Strains cultivation*,” paragraph 1, 103–105.
**Materials and methods**, “*Experimental design and sample collection*,” paragraph 3.
**Results**, “*Growth of MC-producing and MC-free strains*,” paragraph 1, 184–190.
**Results**, “*Metabolite classification and profiling*,” paragraph 2, 202–208.
**Results**, “*DMs of different strains in the same growth phase*,” paragraph 1, 243–244.
**Results**, “*DMs of same strain in different growth phases*,” paragraph 1, 252–253.
**Results**, “*DMs pathway of same strain in the different growth phases*,” paragraph 2, 279–280.
**Discussion**, paragraph 2, 302–310, paragraph 3, 321–342.”

Due to the re-naming of the strains and an added citation, a correction has been made to **Introduction**, paragraph 3. The sentences previously stated:

“*Microcystis* strains may be characterized as ‘Microcystin-producing' (MC-producing strain) or ‘Microcystin-free' (MC-free strain; Davis et al., 2009). It is known that MC-producing strains often coexist with MC-free strains in nature, and their proportions change seasonally (Kurmayer and Kutzenberger, 2003; Lorena et al., 2004; Hu et al., 2016; Islam and Beardall, 2017; Fernanda et al., 2019). Moreover, previous laboratory research suggests that both MC-producing and MC-free strains can be harmful-eliciting damage to mitochondrial function by altering the membrane potential-and that the latter strain may be more toxic than the former (Xu, 2021). Histopathological observations indicate that both MC-free and MC-producing Microcystis aeruginosa induce liver cellular impairments in medaka fish, possibly in association with toxic metabolites (Manach et al., 2018). However, information on other toxic metabolites of MC-free strains is lacking.”

The corrected sentences appear below:

“*Microcystis* strains may be characterized as ‘Microcystin-producing' (MC-producing strain) or ‘Microcystin-free' (MC-free strain; Davis et al., 2009). It is known that MC-producing strains often coexist with MC-free strains in nature, and their proportions change seasonally (Kurmayer and Kutzenberger, 2003; Lorena et al., 2004; Hu et al., 2016; Islam and Beardall, 2017; Fernanda et al., 2019). Histopathological observations indicate that both MC-producing and MC-free *Microcystis aeruginosa* induce liver cellular impairments in medaka fish, possibly in association with toxic metabolites (Manach et al., 2018). Moreover, laboratory research suggests that both high-MC-producing and low-MC- producing strains can be harmful-eliciting damage to mitochondrial function by altering the membrane potential-and that the latter strain may be more toxic than the former (Xu, 2021; Xu et al., 2023). However, information on other toxic metabolites generated by *Microcystis* strains is lacking.”

Due to the re-naming of the strains and an added citation, a correction has been made to **Materials and methods**, “*Experimental design and sample collection*,” paragraph 3. This sentence previously stated:

“Hereafter, we refer to MaE of the MC-producing strain collected at E- and S-phases as MCE and MCS, respectively, while that of the MC-free strain are MCFE and MCFS, respectively.”

The corrected sentence appears below:

“Hereafter, we refer to MaE of the high-MC-producing strain collected at E- and S-phases as MCE and MCS, respectively, while that of the low-MC-producing strain are MCFE and MCFS, respectively. The MC-LR contents of four groups had reached 3.79, 17.42, 2.04, and 7.90 ng/mL for MCE, MCS, MCFE and MCFS, respectively (Xu et al., 2023).”

Due to a change in the references, a correction has been made to **Discussion**, paragraph 2, 304–306. This sentence previously stated:

“Xu (2021) revealed that MaE was toxic to mitochondrial membranes in *D. magna*, and the MC-free strain was more toxic to mitochondrial membrane than a MC-producing strain, and toxicity effects were stronger in S-phase than E-phase cultures.”

The corrected sentence appears below:

“Xu et al. (2023) revealed that MaE was toxic to mitochondrial membranes in *D. magna*, and the low-MC-producing strain was more toxic to mitochondrial membrane than the high-MC-producing strain, and toxicity effects were stronger in S-phase than E-phase cultures.”

The authors apologize for these errors and state that they do not change the scientific conclusions of the article in any way. The original article has been updated.
