# Supplementary material for: Comparative metabolomic analysis of exudates of microcystin-producing and microcystin-free Microcystis aeruginosa strains
Source: Front Microbiol. 2023 Jan 19;13:1075621. doi: 10.3389/fmicb.2022.1075621 (PMC9894096; doi:10.3389/fmicb.2022.1075621)

**Supplementary Figure S1** Score plots of the orthogonal partial least squares discriminant analysis (OPLS-DA) of four groups: MCFE vs MCE (A); MCFS vs MCS (B); MCS vs MCE (C); MCFS vs MCFE (D).

MCE, MC-producing strain at exponential phase; MCFE, MC-free strain at exponential phase; MCS, MC-producing strain at stationary phase; MCFS, MC-free strain at stationary phase.

**Supplementary Figure S2** Values of R2Y and Q2 from the permutation test for the OPLS-DA model of four groups with positive and negative ion modes: 905S vs 905E positive ion mode (A); 526S vs 526E positive ion mode (B); 526E vs 905E positive ion mode (C); 526S vs 905S positive ion mode (D); 905S vs 905E negative ion mode (E); 526S vs 526E negative ion mode (F); 526E vs 905E negative ion mode (G); 526S vs 905S negative ion mode (H).

905E, 526E, 905S, 526S represent MC-producing strain at exponential phase, MC-free strain at exponential phase, MC-producing strain at stationary phase, MC-free strain at stationary phase, respectively.

**Supplementary Figure S1**


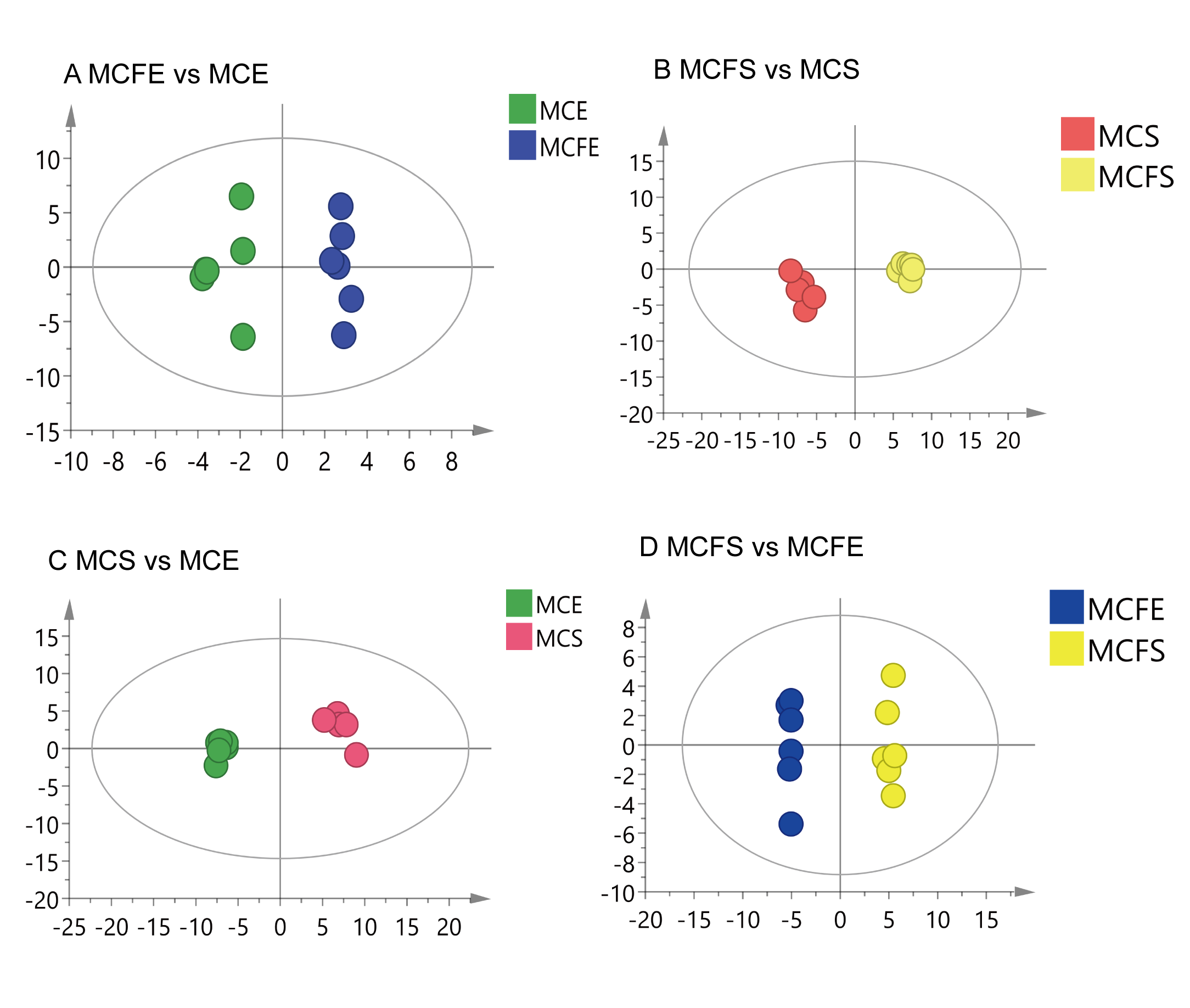


**Supplementary Figure S2**

(A) 905S vs 905E positive ion mode


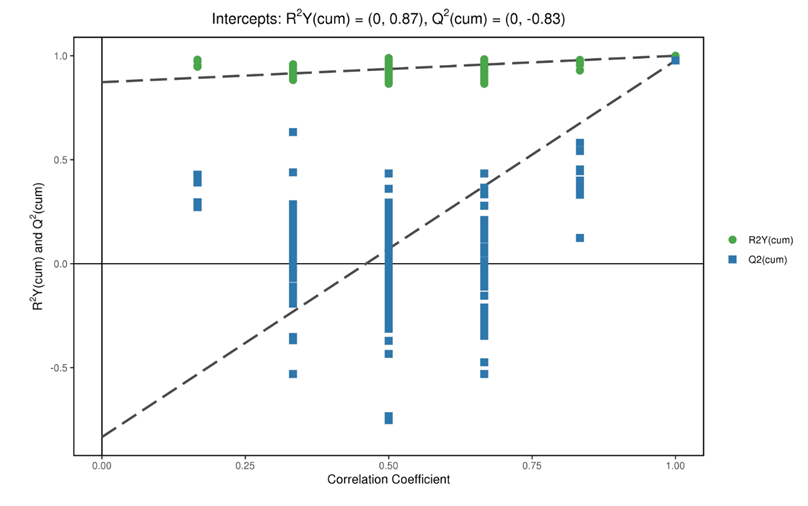


(B) 526S vs 526E positive ion mode


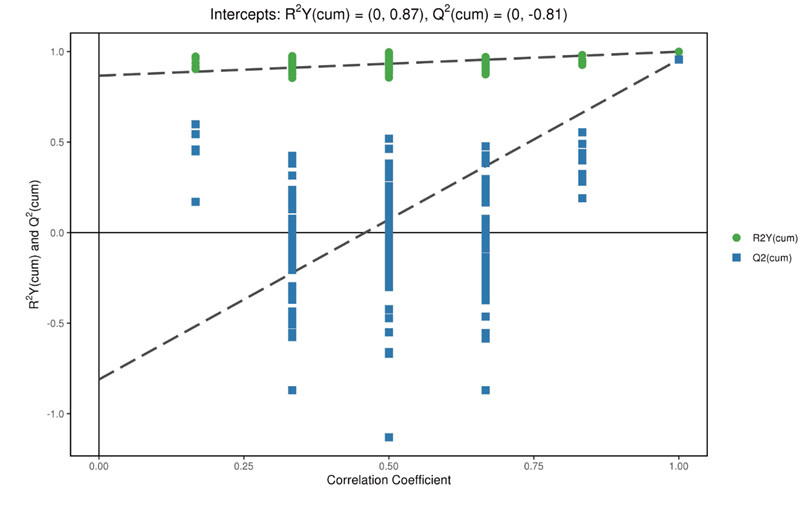


(C) 526E vs 905E positive ion mode


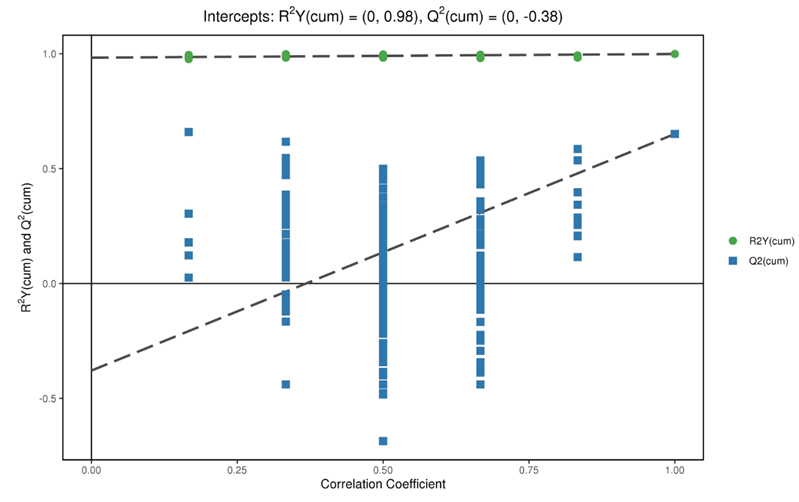


(D) 526S vs 905S positive ion mode


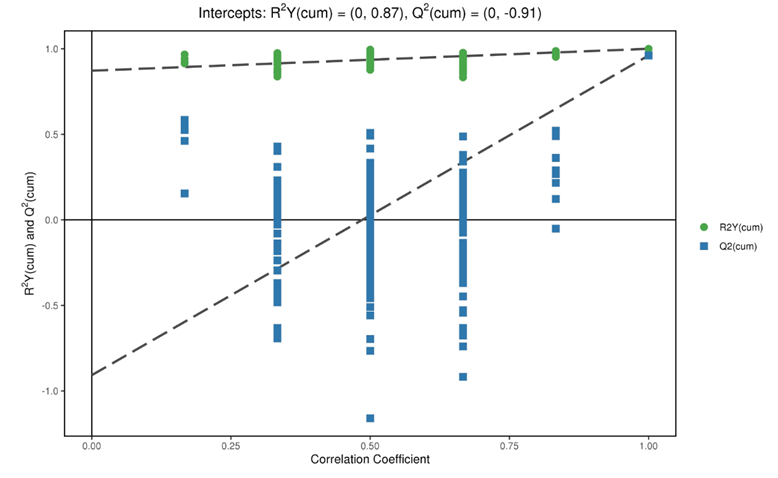


(E) 905S vs 905E negative ion mode


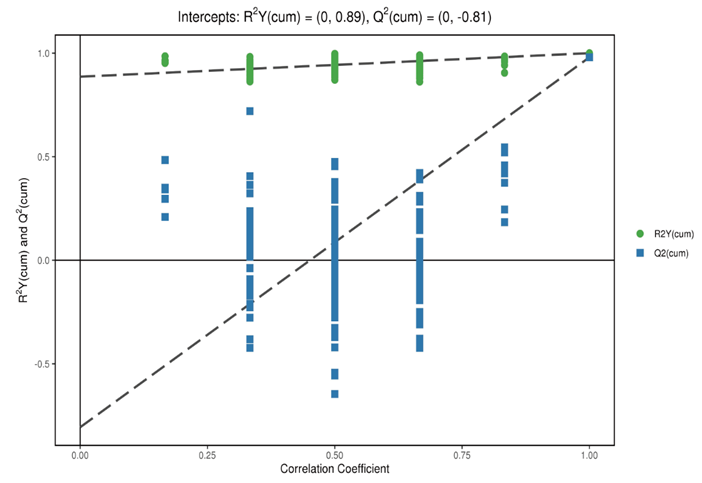


(F) 526S vs 526E negative ion mode


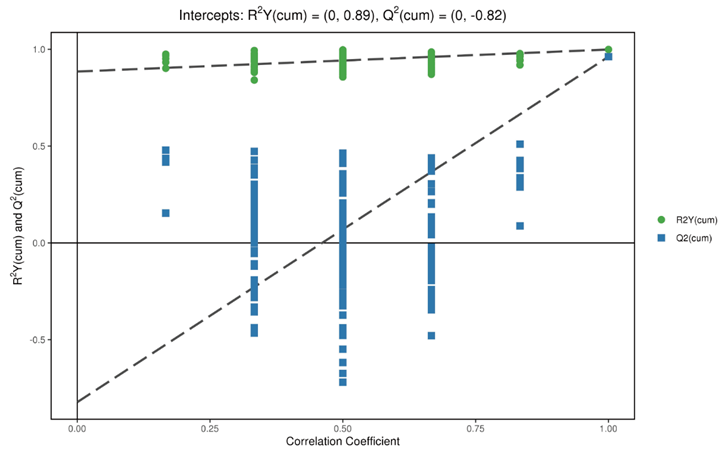


(G) 526E vs 905E negative ion mode


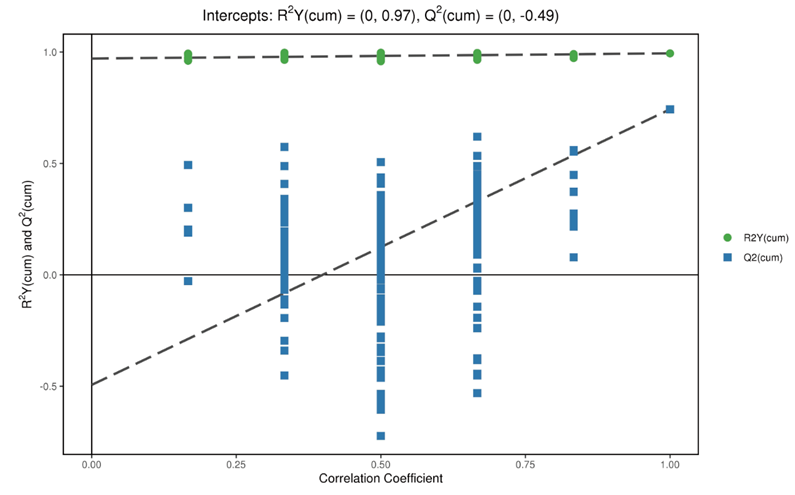


(H) 526S vs 905S negative ion mode


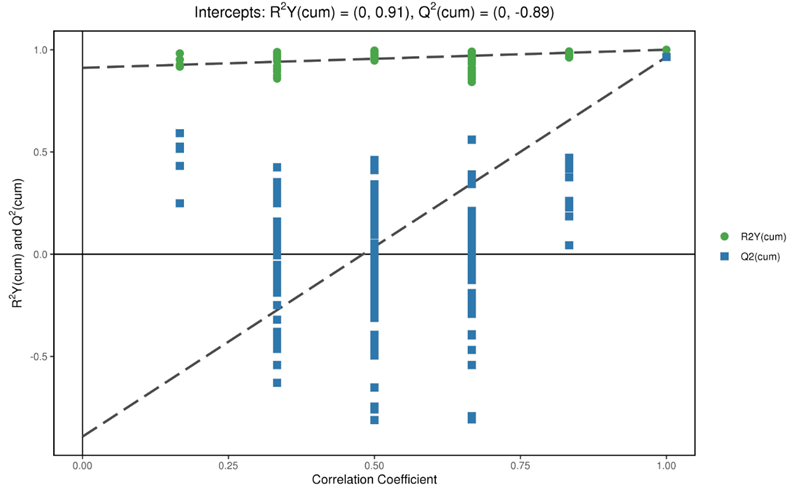


**Supplementary Figure S3** EIC diagram of internal standard in QC and blank samples

1. EIC diagram of internal standard in QC and blank samples (positive ion)


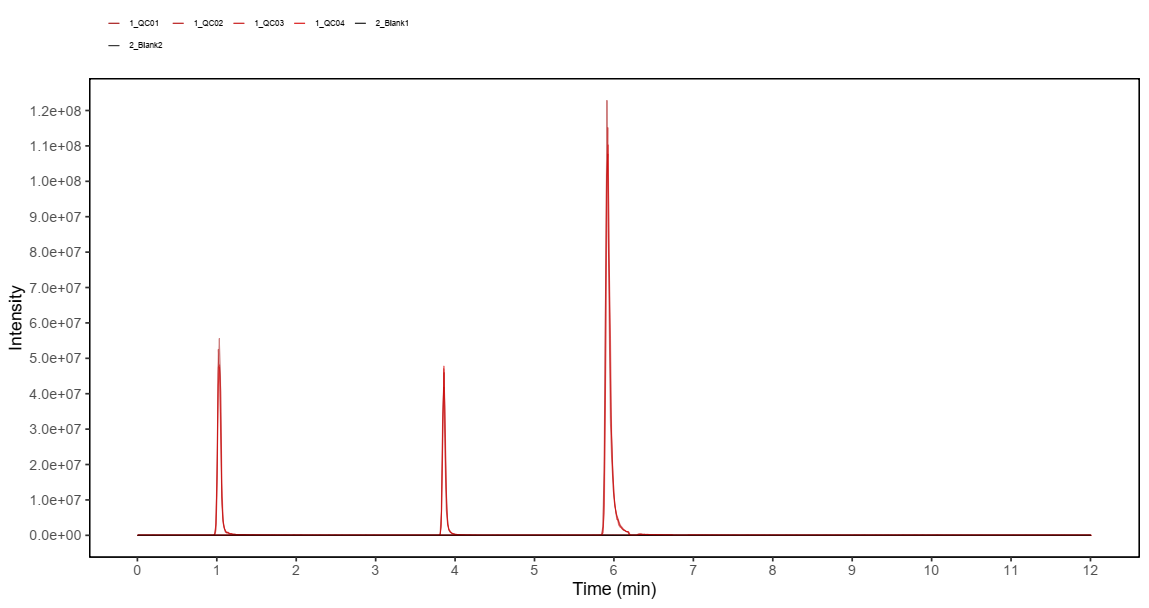


1. EIC diagram of internal standard in QC and blank samples (negative ion)


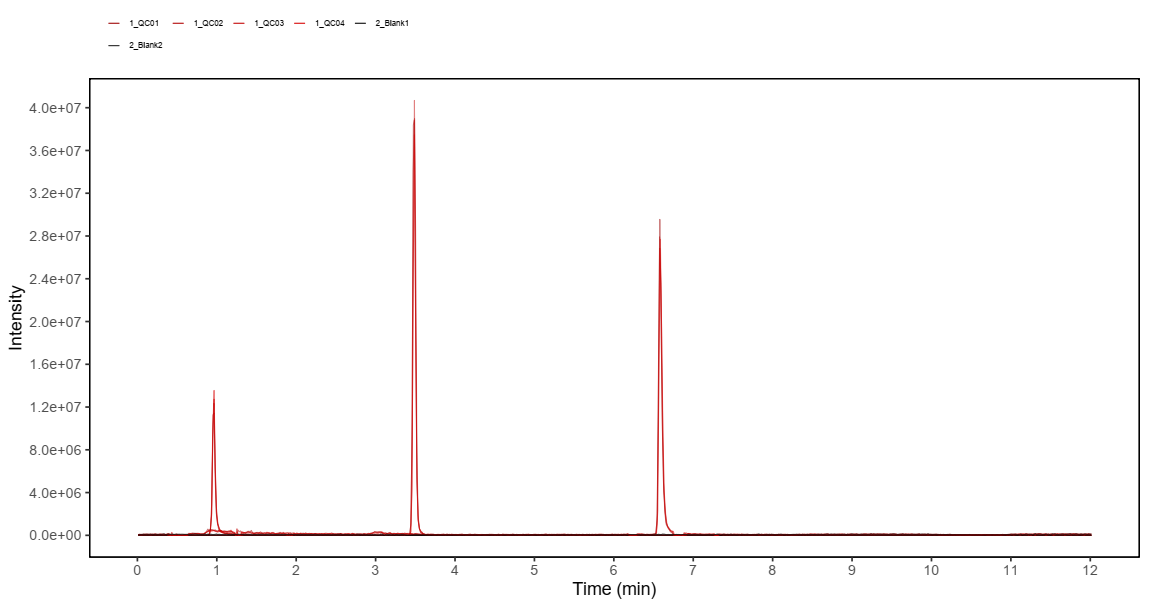

Supplement: Supplementary file 1 [file Data_Sheet_1.doc]
